# A Hepatitis C Virus (HCV) Vaccine Comprising Envelope Glycoproteins gpE1/gpE2 Derived from a Single Isolate Elicits Broad Cross-Genotype Neutralizing Antibodies in Humans

**DOI:** 10.1371/journal.pone.0059776

**Published:** 2013-03-19

**Authors:** John Lok Man Law, Chao Chen, Jason Wong, Darren Hockman, Deanna M. Santer, Sharon E. Frey, Robert B. Belshe, Takaji Wakita, Jens Bukh, Christopher T. Jones, Charles M. Rice, Sergio Abrignani, D. Lorne Tyrrell, Michael Houghton

**Affiliations:** 1 Li Ka Shing Institute of Virology, Department of Medical Microbiology and Immunology, University of Alberta, Edmonton, Canada; 2 Department of Internal Medicine, Saint Louis University School of Medicine, Saint Louis, Missouri, United States of America; 3 Department of Virology II, National Institute of Infectious Diseases, Tokyo, Japan; 4 Copenhagen Hepatitis C Program, Department of Infectious Diseases and Clinical Research Centre, Copenhagen University Hospital, Hvidovre and Department of International Health, Immunology and Microbiology, Faculty of Health Sciences, University of Copenhagen, Copenhagen, Denmark; 5 Laboratory of Virology and Infectious Disease, The Rockefeller University, New York, New York, United States of America; 6 Istituto Nazionale Genetica Molecolare, Milan, Italy; INSERM, France

## Abstract

Although a cure for HCV is on the near horizon, emerging drug cocktails will be expensive, associated with side-effects and resistance making a global vaccine an urgent priority given the estimated high incidence of infection around the world. Due to the highly heterogeneous nature of HCV, an effective HCV vaccine which could elicit broadly cross-neutralizing antibodies has represented a major challenge. In this study, we tested for the presence of cross-neutralizing antibodies in human volunteers who were immunized with recombinant glycoproteins gpE1/gpE2 derived from a single HCV strain (HCV1 of genotype 1a). Cross neutralization was tested in Huh-7.5 human hepatoma cell cultures using infectious recombinant HCV (HCVcc) expressing structural proteins of heterologous HCV strains from all known major genotypes, 1–7. Vaccination induced significant neutralizing antibodies against heterologous HCV genotype 1a virus which represents the most common genotype in North America. Of the 16 vaccinees tested, 3 were selected on the basis of strong 1a virus neutralization for testing of broad cross-neutralizing responses. At least 1 vaccinee was shown to elicit broad cross-neutralization against all HCV genotypes. Although observed in only a minority of vaccinees, our results prove the key concept that a vaccine derived from a single strain of HCV can elicit broad cross-neutralizing antibodies against all known major genotypes of HCV and provide considerable encouragement for the further development of a human vaccine against this common, global pathogen.

## Introduction

HCV is a major global health concern infecting 170 million people worldwide [1]. Replication of the HCV RNA genome is mediated by virus-encoded non-structural protein NS5B, an error prone RNA-dependent RNA polymerase, and the low fidelity of the enzyme has contributed to the high mutagenic rate and broad antigenic diversity of the hepacivirus genus creating a major challenge in developing a global vaccine. Historical therapy using a combination of interferon-alpha and ribavirin has had significant but limited success and while the recent addition of drugs inhibiting a viral protease have increased the overall therapeutic response, this combination exhibits substantial toxicity and more than 30% of patients are not cured [2]. New, highly promising drug cocktails are expected to be available over the next few years and while a complete cure can be envisaged for nearly all treated patients, the high expense and sophisticated clinical care required for these drug combinations makes the prospect of universal delivery very unlikely. Therefore, it remains imperative to develop a global HCV vaccine. However, there are 7 major genotypes of HCV and many hundreds of subtypes distributed globally, with genotype 1a being the most prominent virus in the North America and genotype 1b infecting the most people worldwide [3], [4]. Among all genotypes, there is up to 31–33% nucleotide diversity [4]. Various genotypes of HCV have been shown to have differences in disease outcome and response to antiviral therapy [5], [6]. A global vaccine will therefore have to be effective against this vast diversity of HCV variants and has represented a major challenge.

A small fraction of individuals can spontaneously clear HCV infection leading to the belief that prevention of HCV is possible if a vaccine can elicit similar immune responses [7], [8], [9]. Cellular immunity has been shown to be important to control HCV infection. Depletion of CD4+ or CD8+ T cells has been shown to allow chronic, persistent infection in chimpanzees [10]. On the other hand, the role of antibodies to control HCV infection has been understudied, largely due to the lack of suitable assays for neutralizing and cross-neutralizing antibodies, until recently [11], [12], [13], [14], [15]. Cross-neutralizing antibodies can be isolated from chronically-infected patients [16], [17], [18] but only years after the original infection when virus-specific cellular immune responses are already blunted [17]. Despite the failure of these antibodies to eradicate chronic infection, there is evidence that they are actively driving evolution of the viral envelope glycoproteins suggesting they are partially controlling infection [19]. More recently, studies have demonstrated a correlation between the presence of neutralizing antibodies and the clearance of acute infection without the development of chronic, persistent infection [9], [20], [21]. Furthermore, cross-neutralizing antibodies have been shown to confer protection in passively-immunized SCID mice transplanted with human hepatocytes [16], [22].

All successful viral vaccines developed to date have been based on the induction of neutralizing antibodies [23], [24] usually targeting the virion surface proteins. An important function of these proteins is to interact with cellular receptors to mediate cell entry and to fuse with host membranes during uncoating [25]. Neutralizing antibodies have been identified in natural HCV infections targeting these proteins [26]. Our earlier work has shown that a recombinant gpE1/gpE2 HCV vaccine is immunogenic in guinea pigs [27] and chimpanzees [28] and has been shown to induce protective immune responses in the latter model against experimental challenge with either homologous or heterologous genotype 1a HCV strains [28]. Vaccinated chimpanzees had a significantly reduced rate of chronicity following experimental challenge and some animals were even sterilized against homologous virus challenge [29], [30], [31]. The gpE1/gpE2 antigen was derived from strain HCV1 of genotype 1a, the first identified HCV genome [32]. A phase I dose-ranging clinical trial has been conducted to test the safety and immunogenicity of this vaccine in healthy volunteers [33]. All volunteers elicited antibodies against the glycoproteins gpE1/gpE2 as measured in EIA formats [33] and the vaccine was effective in inducing strong T-helper responses to the vaccine [33]. Further studies have shown that the vaccine induced antibodies targeting known neutralizing epitopes and the sera of selected vaccinees prevented *in vitro* infection by HCV derived from genotypes 1a and 2a [34], [35]. Evidence for cross-neutralization of some diverse genotypes was derived from vaccinated animals [22], [27], [28].

In this study, antisera from the phase I clinical trial was assessed for cross neutralizing activity against representatives of all seven major genotypes of HCV that occur globally. Very broad cross-neutralization activity was evident but not all genotypes were neutralized with similar efficiencies. We conclude that this vaccine can elicit cross-neutralizing antibodies in human that target epitopes that are highly conserved among all major genotypes of HCV. When combined with the demonstrated efficacy of this vaccine in the chimpanzee model [30], the current findings strongly encourage the further development of this and related vaccine candidates.

## Results

### Recombinant gpE1/gpE2 vaccine elicits neutralizing antibodies

**Figure 1:**
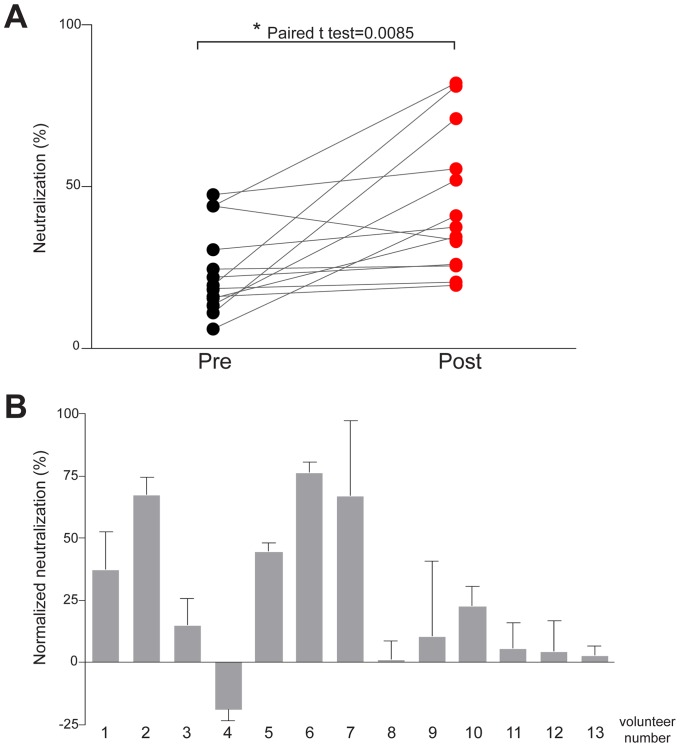
Human antisera neutralizes heterologous 1a infectivity in cell culture. Pre or post vaccination sera were incubated with chimeric H77C/JFH-1 HCVcc followed by infection of naïve huh7.5 cells. Representatives of two independent experiements performed in triplicates are shown. The number of infected cells was quantitated by immunostaining using anti-NS5A antibodies 48 hour post-infection. (A) Neutralization activity was calculated using a negative control lacking serum (0%) and anti-CD81 antibody as a positive control (%). “Pre” sera were collected prior to vaccination and “post” sera were collected 2 weeks post-third immunization [33]. Grey line connects the neutralization activity of pre- and post- vaccination of the same volunteer. The paired t test score of mean neutralization activity between pre- and post-groups is shown indicating a significant difference. (B) Neutralization activity of post-vaccination sera was normalized using pre-vaccination sera of the same individual. The neutralization activities of three volunteers' sera within this group were not shown due to inconsistent results from two independent experiments.

During the phase I dose-ranging clinical trial testing the recombinant gpE1/gpE2 vaccine in healthy volunteers, maximal anti-gpE1/gpE2 EIA antibody titers were observed at two weeks post-third immunization [33]. Accordingly, we examined the neutralizing activity of volunteers' sera collected at two weeks post third vaccination using the highest dose (100ug) of antigen in this study. Chimeric virus encoding core, gpE1, gpE2, p7 and NS2 genes from heterologous H77C (genotype 1a) in the backbone of JFH-1 genome has been produced to allow the study of genotype 1a specific entry [3]. This heterologous 1a chimeric virus was pre-incubated with dilutions of volunteers' sera then added to cultured hepatoma Huh7.5 cells and the subsequent level of infection was quantified 2 days post-infection. As shown in Figure 1A, post-vaccination sera showed significant neutralization of the heterologous 1a chimeric virus, with 5 of 13 sera being able to neutralize over 50% of the virus, two of which neutralized up to 80% of viral infectivity. Components in human sera, such as apolipoproteins, have been shown to have non-specific effects on virus entry and therefore may have contributed to the variable background in the pre-vaccination samples [36], [37]. In order to control for individual differences in serum components, the neutralization activity of post-vaccination sera was normalized using the neutralization activity of the pre-vaccination sera from the same individual (Figure 1B). This analysis showed that 92% (12/13) individuals elicited significant neutralization activities, 5 of which (volunteers 1, 2, 5, 6 and 7) showed higher neutralizing activity compared to others within the group. These data showed the vaccine was capable of eliciting neutralizing antibodies against heterologous 1a virus infection. Importantly, volunteer 4 appeared to display somewhat less virus neutralization after vaccination which is consistent with an possible enhancement effect of the vaccine. Further work is needed to ascertain if this effect is truly due to vaccine enhancement in this individual or due to a high, variable background in this particular individual combined with a low level of neutralization elicited by the vaccine in volunteer 4.

### Vaccine induced antibodies confer broad cross-genotype neutralization *in vitro*

**Figure 2:**
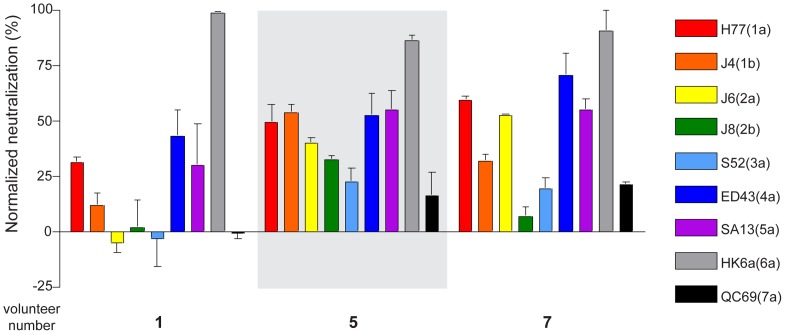
Human antisera cross-neutralizes all 7 major HCV genotypes. Sera of volunteers 1, 5 and 7 were tested for neutralization activity against chimeric 1a (H77C), 1b (J4), 2a (J6), 2b (J8), 3a (S52), 4a (ED43), 5a (SA13), 6a (HK6a) or 7a (QC69) HCVcc [3]. Virus neutralization assays were performed using pre- and post- vaccination sera at a concentration of 1 in 50. Levels of neutralization activity of post-vaccination sera were normalized with the activity of pre-vaccination sera. Representative of two independent experiments performed in triplicate are shown.

We chose sera from volunteers 1, 5 and 7, due to their high neutralization activities among assayed volunteers, to test for cross-genotype neutralization activity using chimeric HCVcc encoding core, gpE1, gpE2, p7 and NS2 genes derived from representative strains of all 7 major genotypes that occur globally [3]. In Figure 2, volunteers 5 and 7 showed a broad range of neutralization activity against viruses of all 7 major genotypes. The profiles of broad cross neutralization activity are very similar using both sera but in both cases (and with volunteer 1), less cross-neutralization was observed against 2b, 3a and 7a viruses. This indicates that while there must be a neutralizing epitope(s) that is highly conserved across all clades of HCV, genotype-specific neutralizing epitopes may also be present.

Interestingly, all sera tested showed strong neutralization activity against the chimeric virus HK6a/JFH-1. There are two adaptive mutations in the glycoprotein region, F350 within gpE1 and N417 within gpE2. The N417 is a highly conserved residue among all genotypes and mutation of this site leads to elimination of a N-glycosylation site [3]. These mutations appear to confer higher sensitivity to neutralization (Figure 2). This is consistent with other data showing that various HCV antibodies neutralize the tissue culture-adapted HK6a efficiently despite showing low neutralization activity against other genotypes of HCV (data not shown).

**Figure 3:**
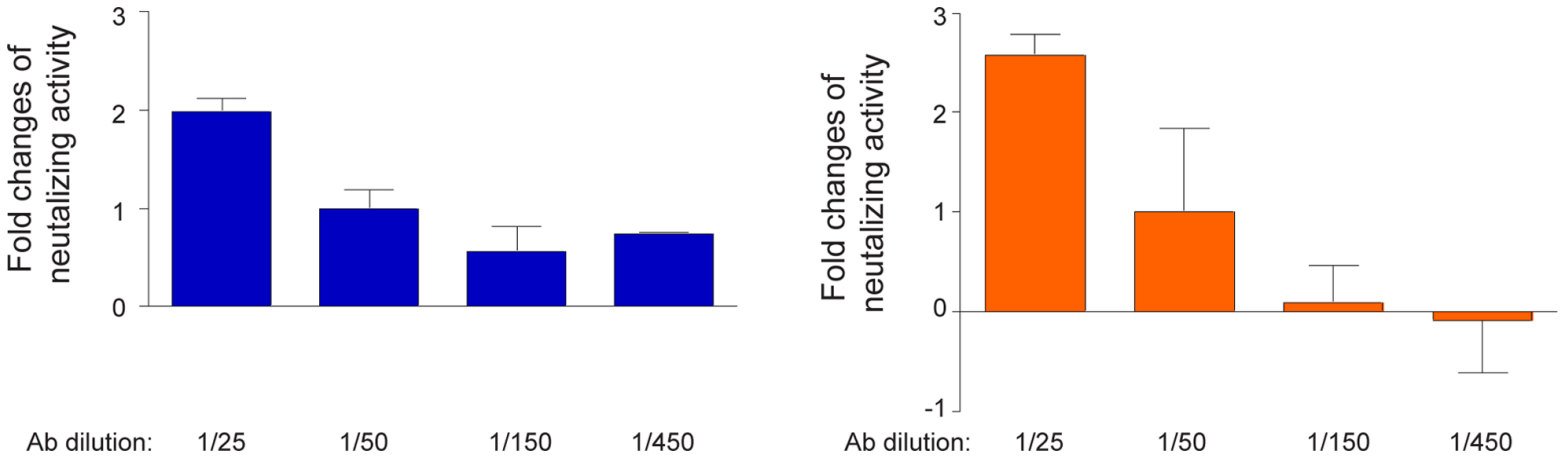
Human antisera neutralizes HCVcc in a dose-dependent fashion. Serum of volunteer 5 was tested for neutralization activity in various doses against 1a (H77C, blue) or 2a (J6, orange) chimeric HCVcc. Sera at indicated dilution were incubated with 100 TCID50 of HCVcc. The level of infection was monitored after staining using NS5A antibodies. Results of an experiment preformed in triplicates are shown. Fold changes of neutralization activity is shown compared to serum using at 1 in 50 dilution.

We observed a dose-dependent neutralization with increasing amount of sera (Figure 3). Increasing the antiserum concentration two-fold (to a 1∶25 dilution) resulted in a significant enhancement of neutralization activity compared with the standard dilution used in the other figures of this paper (1 in 50 dilution). This enhancement was observed against infection of both genotype 1a and genotype 2a viruses.

### The Neutralization activity of human antisera is mediated by immunoglobulin

**Figure 4:**
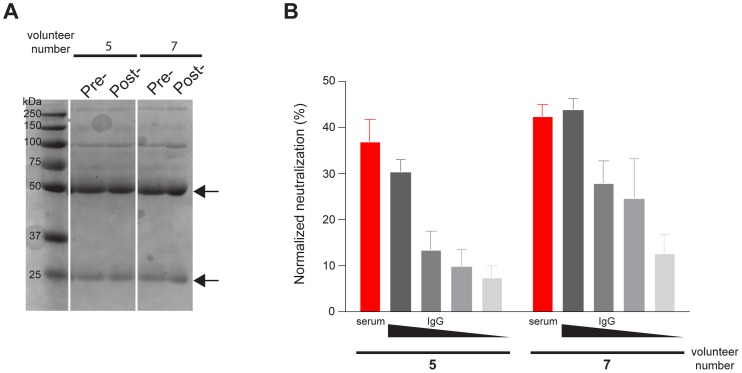
Neutralization of human antisera is mediated by immunoglobulin. Immunoglobulin was purified from volunteers' pre- or post- vaccinated sera. (A) 3 μg total protein of purified immunoglobulin of volunteers 5 and 7 were separated by SDS-PAGE followed by coomassie staining for visualization. Heavy and light chains of purified immunoglobulin are marked with arrows. (B) The starting concentration of purified immunoglobulin was used at 1 in 5 (by volume) followed by three 3-fold dilutions (i.e. 1 in 15, 1 in 45 and 1 in 135) in the neutralization assay using chimeric 1a H77C/JFH-1 HCVcc (grey). The neutralization activities of the post- vaccinated samples were normalized against pre-vaccinated samples of the same individual at the indicated dilution. As comparison, the neutralization activity of sera using at 1 in 50 dilution is shown (red). During the purification, the recovered immunoglobulin were diluted 10 times in the procedure, therefore, 10 times more in volume of purified immunoglobulin (1 in 5 dilution) was used compared with sera (1 in 50 dilution) (see materials and methods).

The sera of the vaccinated volunteers was shown to have neutralization activity inhibiting HCV infection. We wanted to examine if this inhibition was antibody-mediated. Immunoglobulins were purified from antisera and tested for neutralization activity. The isolation achieved over 90% purity of immunoglobulin monitored by SDS-PAGE (Figure 4A). Subsequent neutralization assays revealed that the purified-immunoglobulin accounts for the majority of the neutralization activity, since the level of neutralization activity was comparable between serum and purified immunoglobulins. As expected, the neutralization activity of the purified immunoglobulin was diminished upon dilution of the amount of IgG added to the assay (Figure 4B).

## Discussion

In this study, the neutralization activities of sera from human volunteers vaccinated with a recombinant HCV gpE1/gpE2 vaccine in a phase I clinical trial were evaluated. The vaccine can induce cross neutralizing activity against heterologous 1a challenge *in vitro* (Figure 1). The neutralization activity of sera was mediated by neutralizing antibodies (Figure 4) and 92% of post-vaccinated sera showed evidence of neutralization activity against the 1a genotype which predominates in Canada and the USA. Furthermore, two of the three tested sera showed broad cross-neutralizing activity against representative viruses from all 7 HCV major genotypes that are known to occur globally (Figure 2). This shows that despite previous concerns about HCV envelope glycoprotein vaccines being only able to elicit isolate-specific neutralization, a vaccine derived from envelope glycoproteins of a single genotype can elicit a broad, cross-genotype neutralizing response. The tested sera showed better neutralization activities against viruses of genotypes 1a/b, 2a, 4a, 5a and 6a, as compared with genotypes 2b, 3a and 7a, indicating that a cocktail of diverse antigens may constitute an optimal global vaccine, although it remains to be determined if antigens from a single strain can still confer adequate global protection.

The recombinant gpE1/gpE2 vaccine has been shown earlier to elicit a significant level of cross neutralization antibodies in the chimpanzee model [28] and also to be efficacious at reducing the incidence of chronic infection following experimental challenge with either homologous or heterologous viral strains [30]. The breadth of neutralizing antibodies elicited in chimpanzees was similar to our finding reported here, with the vaccinated sera of chimpanzees being more effective at neutralizing virus of genotypes 1a, 4a 5a, 6a compared with genotype 2a and 3a (genotype 7a was not tested) [28]. Although human antisera only partially neutralized HCVcc *in vitro*, lower dilutions of serum resulted in greater neutralization (Figure 3). Cross-neutralizing antibodies isolated from chronically infected patients or by molecular cloning have been shown to protect humanized mice against heterologous viral infections [16], [22]. It will be important to test if the vaccine-induced antibodies reported here exhibit a similar protective potency although this would seem feasible.

The vaccine was successful at inducing E1E2-reactive antibodies [33], but not all sera have strong neutralization activity as shown in this study and elsewhere [35]. Limited characterization of these vaccine antibodies has been reported [34]. Viral neutralization may be facilitated by a strong avidity of antibodies to previously identified neutralizing eptitopes [35], although new, unidentified neutralization epitopes may exist. We are performing research aimed at mapping the neutralization epitopes targeted by this vaccine candidate in humans. Since broad cross-neutralization has been observed, at least one cross-neutralizing epitope must be highly conserved throughout all genotypes of HCV despite the presence of considerable genetic heterogeneity elsewhere in the viral genome. Broad, cross-neutralizing monoclonal antibodies have been isolated previously [26] and it remains to be determined which of these antibody epitopes are also targeted by this vaccine.

In this study, we have used chimeric viruses derived in cell culture to identify cross-neutralizing antibodies. It has been shown that the physical properties of cell culture-derived virus are different from animal-derived virus due to differences in lipid composition [38]. The term “lipo-viro particle” has been used to reflect the close association of HCV virus with apolipoproteins which may affect the cell entry process [39], [40]. Further studies are needed to directly examine the neutralization activity of human vaccinee antisera against virus derived from infected chimpanzees and humans. Dreux *et al* have reported a negative impact of human serum components on the activity of neutralizing antibodies against HCV pseudoparticles [41]. However, we have observed similar neutralization activity when immunogloblin was purified with high efficiency from our human antisera and tested at amounts equivalent to the original volume of serum. This would indicate that in human vaccinees, no such inhibition of neutralization of HCVcc is detectable. It was also of interest to detect very effective neutralizing antibodies against the chimeric virus bearing genotype 6a envelope glycoproteins since this particular chimeric virus is the only one containing adaptive mutations within the envelope glycoprotein coding region [3]. It remains to be determined which of these mutations mediates this enhanced sensitivity to neutralization.

The data shown in this work indicates that a vaccine derived from a single strain of HCV is capable of eliciting broad cross-neutralizing antibodies implying that there must be a highly-conserved neutralization epitope(s) within the highly variable gpE1/gpE2 envelope glycoproteins. The cross-neutralization titers have so far been detected in only a minority of vaccinees and tended to be low and although it is unknown what titers actually mediate vaccine efficacy, it will be important in future to attempt to enhance the immunogenicity of the vaccine by further modifications to the antigens, adjuvant and formulation (work in progress). When combined with previous data demonstrating the protective efficacy of this vaccine in the chimpanzee model, these data offer considerable encouragement for the eventual production of an efficacious global vaccine to prevent the development of chronic, persistent infection and associated disease in exposed individuals.

## Materials and Methods

### Cells and viruses

Huh7.5 cells were cultured in DMEM supplemented with 10% FBS, 0.1 mM NEAA and 100 μg each of penicillin and streptomycin as described [14].

Cell culture derived HCV (HCVcc) are produced using previously described protocol [14]. Cells were washed twice with ice cold PBS and subsequently resuspended to 1.5×10^7^ cell/ml. 400 μl of the cell suspension were mixed with 5 μg *in vitro* transcribed RNA encoding HCV genome in 2 mm gap electroporation curvettes. 5 pulses of 860 V (99 μs, 1.1 s interval) were delivered using the ElectroSquare Porator ECM 830 (BTX, Holliston, MA). Post-electroporation, cells were incubated at room temperature for 10 minutes before plating. Pre-cleared media was collected as virus stocks either 3 or 4 day post-electroporation. Virus titer (50% tissue culture infectious dose (TCID50)) was determined by limited dilution as described [14].

### Volunteers' sera and neutralization assay

All sera were acquired from a completed phase I randomized, double-blinded, placebo-controlled study assessed the safety and immunogenicity of HCV E1E2/MF59C.1 (DMID01–012), approved by the Saint Louis University Institutional Review Board (IRB #15719) [33]. All volunteers' sera of pre- and post- vaccination were heat inactivated at 56°C for 1 hour.

1×10^4^ huh 7.5 cells per well were seeded on poly-lysine coated 96 well plates, 1 day prior to infection. For infection, 100 TCID50 HCVcc were premixed with heat inactivated sera diluted at 1 in 50 (by volume), for 1 hour at 37°C followed by adding to cells. 12 hour post-infection, the antibody-virus inoculum was replaced with fresh culture media. Cells were then fixed 48 hours post-infection with methanol using previously described methods [14]. The amount of infection was quantitated by counting the number of NS5A-positive foci detected using mouse monoclonal NS5A antibody (9E10) [14]. The foci were detected and counted using a CTL S6 immunospot analyzer (CTL, Cleveland Oh) as described [42]. The percentage of neutralization was reported by comparison with no serum control or normalized with pre-vaccination serum as described in text. The neutralization activity was calculated using the following formula: % neutralization =  (pre-post)/pre ×100% where pre/post represent the number of NS5A-positive foci done after incubating with either the pre- or post- vaccination sera as described in text.

### *In vitro* RNA transcription

Plasmids encoding chimeric HCV genomes representing all major genotypes (1–7) have been described [3]. DNA templates were generated by linearizing plasmids using *Xba* I and infectious RNA were generated using T7 RiboMAX large scale RNA production system (Promega, Madison, WS). RNA was subsequently purified using the RNeasy mini kit (QIagen, Hilden Germany).

### Immunoglobulin purification

Immunoglobulin was purified using the Melon gel IgG spin purification kit (Thermo Scientific, Rockford IL). The purified immunoglobulin was diluted 10 times in volume compared to the starting material, i.e. 500 μl of purified immunoglobulin was recovered at the end by starting with 50 μl of serum. Therefore, ten times more (by volume) of purified immunoglobulin (as compared with the original serum) was used to compare the neutralization activity between the purified immunoglobulin and the serum. The purity of isolated immunoglobulin was monitored by SDS-PAGE followed by coomassie blue staining. The purity of immunoglobulin using this method was higher than 90%.
